# Liposomal Encapsulation Enhances *In Vivo* Near Infrared Imaging of Exposed Phosphatidylserine in a Mouse Glioma Model

**DOI:** 10.3390/molecules181214613

**Published:** 2013-11-26

**Authors:** Liang Zhang, Dawen Zhao

**Affiliations:** Department of Radiology, The University of Texas Southwestern Medical Center, 5323 Harry Hines Boulevard, Dallas, TX 75390, USA

**Keywords:** phosphatidylserine (PS), liposomal nanoprobe, near-infrared (NIR) optical imaging, tumor vasculature, glioma

## Abstract

We have previously demonstrated that exposed phosphatidylserine (PS) on tumor vascular endothelial cells is highly tumor specific, and development of the PS targeted near infrared (NIR) optical probe enables successful *in vivo* optical imaging of U87 gliomas in a mouse model. Liposomes have been widely used as a nanovector for delivery of chemotherapeutics and imaging contrast agents due to their high payload and longer circulation time. In the current study, we have fabricated PS-targeted liposomal nanoprobes encapsulating a NIR dye, IRDye^®^ 800CW, aiming to enhance PS-targeted tumor imaging. Hydrophilic 800CW dye was packed into the core of polyethylene glycol (PEG)-coated liposomes functionalized with F(ab’)_2_ fragments of PGN635, a fully human monoclonal antibody that binds PS. As expected, *in vivo* dynamic NIR imaging revealed significantly improved tumor/normal contrast (TNR = 20 ± 3; *p* < 0.01) of subcutaneous U87 gliomas in mice after injection of the liposomal nanoprobes. Markedly enhanced TNR was observed after the tumors were irradiated to increase PS exposure (TNR = 48 ± 6; *p* < 0.05). Intriguingly, the liposomal nanoprobes, PGN-L-800CW showed distinct biodistribution and pharmacokinetics compared to the 800CW-PGN probes used in our previous study. Our data further suggest the usefulness of PS-targeted imaging probes for sensitive tumor detection and the potential of utilizing liposomal platform for glioma theranostics.

## 1. Introduction

Development of tumor-specific imaging probes is critical for improved tumor targeting and thus enhanced localization of tumors by diagnostic imaging. Cell surface-exposed phosphotidylserine (PS) is an attractive target for molecular imaging. In normal cells, the phosphatidylserine (PS) is asymmetrically distributed across the plasma membrane with essentially all the PS located in the cells’ inner membrane leaflet. However, loss of PS asymmetry occurs during apoptosis and necrosis. PS exposure on the apoptotic cells is irreversible and the cells are subject to phagocytosis by antigen-presenting cells (APCs) [1]. Much interest has been generated in developing molecular imaging probes that bind to the exposed PS in order to noninvasively monitor the response of cancer treatment. Annexin V (A5) is the PS binding ligand that is most widely used for this purpose [2,3].

Recent studies by Thorpe’s lab have demonstrated that the oxidative stress within tumor microenvironment causes redistribution of phosphatidylserine (PS) from the inner to the outer membrane leaflet of tumor endothelial cells [4,5]. The vascular endothelium in normal tissues, even in those highly angiogenic ovarian blood vessels during ovulation, lacks exposed PS. Unlike the apoptotic cells, the PS exposed endothelial cells are viable and not subject to apoptotic processes [6,7]. These cells that are not co-stained by anti-active caspase 3 antibody can resume growth and reestablish phospholipid asymmetry, which enable them to evade immune surveillance [8,9]. He *et al*. further investigated the differential sensitivity between tumor vascular endothelial cells and tumor cells in response to a total of 10 Gy radiation in lung A549 tumors. A 22% increase in PS exposure was observed on tumor vasculature 12 h after radiation while there was minimal change in the number of PS-exposed tumor cells [9]. Thus, the luminal surface-exposed PS may be a useful biomarker of tumor vasculature.

Utilizing PGN635, a novel human monoclonal antibody that binds PS, we have recently developed an optical imaging probe by conjugating the antibody with near infrared dye, IRDye^®^ 800CW. After systemic injection of 800CW-PGN635, *in vivo* NIR imaging successfully detected significant uptake of the probes by U87 glioma xenografts in mice [10]. Both *in vivo* imaging and histological validations proved that 800CW-PGN635 is a highly tumor-specific probe for optical imaging.

However, it has been noticed that such imaging probes of targeting ligand-fluorescence dye may have significant drawbacks [11]. The limited number of ligands and fluorophores per conjugate and high probability of interactions of the probes with blood contents may affect maximal targeting of the probes to achieve optimal imaging contrast. Liposomes-based nanocarriers have been widely applied for improved molecular transport [12]. The unique features of liposomes that make them suitable for tumor-targeted imaging include: (1) their ability to hold a large contrast agent payload; (2) the protective bilayer shielding the enclosed molecules from interaction with contents of the blood stream; (3) surface coating with polyethylene glycol (PEG)-polymers prolonging blood circulation time, resulting in enhanced permeability and retention (EPR) of contrast agents in tumor; and (4) the large surface area allowing increased number of ligand binding sites [12,13,14,15].

In the present study, we have attempted to improve the ability of PS-targeted optical imaging probes for sensitive imaging of glioma by using a liposomal nanodelivery system. We developed PS-targeted liposomal nanoprobe, PGN-L-800CW, containing near infrared dye, IRDye^®^ 800CW. The F(ab’)_2_ fragments of PGN635 were bound to the distal terminus of PEG chains that were coated on the surface of liposomes. After characterization of the chemical and physical properties of the nanoprobes and confirmation of their *in vitro* binding specificity, we investigated the *in vivo* behavior of PGN-L-800CW in subcutaneous U87 glioma bearing mice, the same model as used in our previous study, which enabled a direct comparison between the two probes.

## 2. Results and Discussion

### 2.1. Characteristics of PGN-L-800CW

**Figure 1:**
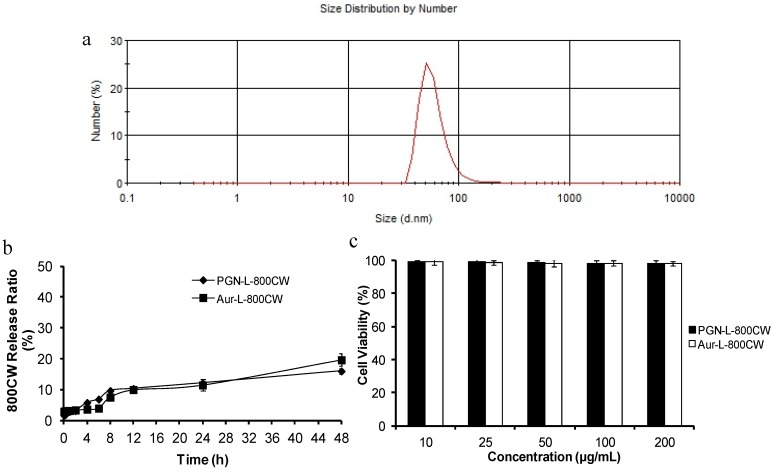
Characterization of the liposomal nanoprobes. (**a**) A size distribution curve obtained by dynamic light scattering (DLS) analysis indicates an average hydrodynamic size of PGN-L-800CW = 65 nm; (**b**) Release rates of 800CW from liposomes in PBS containing 10% serum oscillated with a shaker at a rate of 100 times per minute at 37 °C. Release rate of 800CW was negligible. c. Minimal cytotoxicity of ABAE endothelial cells was observed by trypan blue staining after incubation with the liposomal nanoprobes.

The F(ab’)_2_ fragments of PGN635 were used to functionalize the distant terminus of PEG-polymer on the surface of liposomes. Whole antibodies that expose their constant regions (Fc) on the liposomal surface are more susceptible to Fc-receptor-mediated phagocytosis by the mononuclear phagocyte system. Therefore, an F(ab’)_2_ fragment was selected. The liposomal nanoprobes PGN-L-800CW and their control Aur-L-800CW were characterized with respect to particle size, charge, encapsulation efficiency and antibody coupling efficiency, as illustrated in Figure 1 and Table 1. Stability and toxicity studies were also performed, showing that the liposomes were stable in serum for at least 48 h and minimally toxic to adult bovine aorta endothelial (ABAE) cells.

**Table 1:** Characteristics of liposomal nanoprobes.

**a. Encapsulation efficiency of 800CW in the liposomes**		
Liposomes	800CW (%)	
PGN-L-800CW	55.61 ± 2.21	
Aur-L-800CW	51.16 ± 3.39	
**b. Modifying efficiency of antibody on the liposomes**		
Liposomes	Modifying rate (%)	
PGN-L-800CW	82.66 ± 9.98	
Aur-L-800CW	80.33 ± 10.01	
**c. Size and electrical potential**		
Liposomes	Mean size (nm)	Zeta potential (mV)
PGN-L-800CW	65.59 ± 3.98	−3.52 ± 0.46
Aur-L-800CW	67.71 ± 4.06	−3.49 ± 0.87

Data was represented as the mean ± standard deviation. Each assay was repeated in triplicate.

### 2.2. *In Vitro* PS-Targeting Specificity of PGN-L-800CW

**Figure 2:**
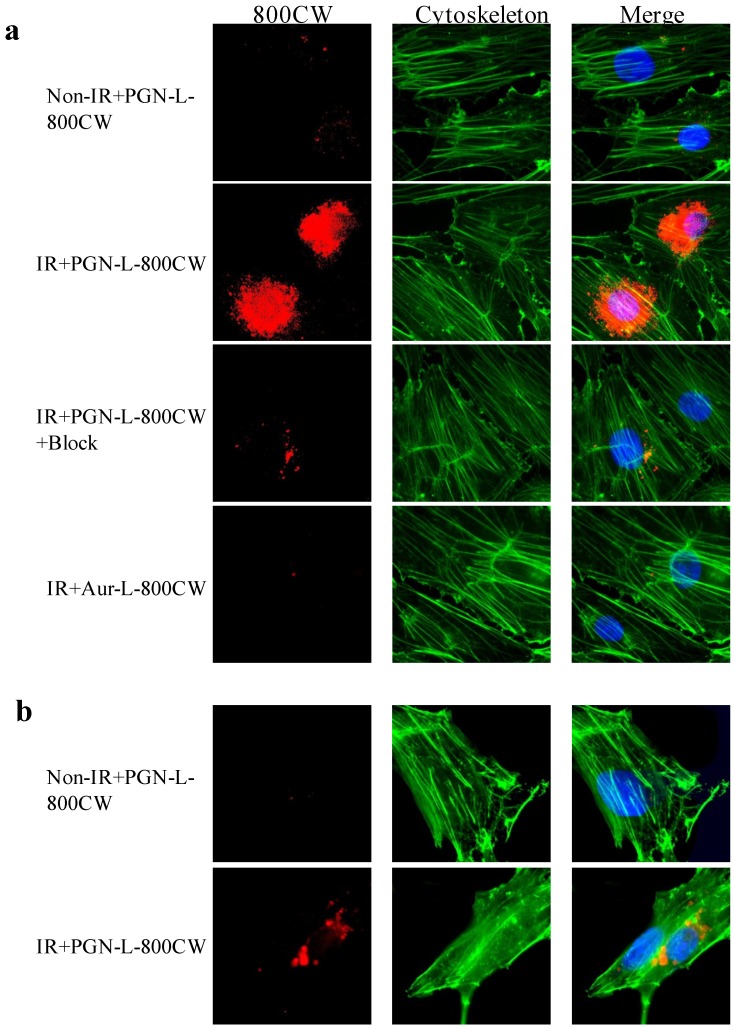
*In vitro* specificity studies of PGN-L-800CW. (**a**) Immunocytochemistry of ABAE endothelial cells pretreated with/without radiation (IR) of a single dose of 6 Gy. Twenty-four hours later, the cells were incubated with either PGN-L-800CW or the control Aur-L-800CW for 1 h before fixation and subsequent staining. Merged fluorescence images showed minimal 800CW (red) signals from non-irradiated cells or Aur-L-800CW treated irradiated cells. By contrast, massive 800CW signals were seen intracellular in the PGN-L-800CW treated irradiated cells. Prior treatment with the full length PGN635 blocked majority of 800CW signals; (**b**) Similar results were observed for the U87 glioma cells that IR induced PS exposure and internalized 800CW signals while minimal 800CW was detected in the non-IR treated cells. Note: green, cytoskeleton actin stained by phalloidin; blue: DAPI stained nuclei.

Under normal culture conditions, there is essentially no PS-exposed ABAE or U87MG cell. To induce PS exposure, ABAE cells and U87 glioma cells were irradiated 24 h earlier with a single dose of 6 Gy to induce exposure of PS on the outer membrane of the cells. Immunocytochemical staining revealed specific binding of PGN-L-800CW to PS exclusively in the irradiated cells, but not in the non-irradiated cells (Figure 2). The control Aur-L-800CW did not bind to PS-exposed cells (Figure 2).

Specificity of PGN-L-800CW was further confirmed by pre-incubating with non-labeled PGN635 to block the binding of PGN-L-800CW (Figure 2). One interesting observation in the current study was that PGN-L-800CW predominantly localized intracellularly. This is in contrast to previous reports that the PS antibody-β2GP1-PS complex remains on external cell membrane for a few days before the macrophages recognize and clear them out [4,16]. Likewise, conjugates of the antibodies with fluorescence dye in our previous study were found not to be internalized [10,17].

The distinguishing feature of liposomal nanoprobes may result from the fusion of the lipid layers between liposomes and cells after PGN635 bound to PS on the cell membrane. In fact, it has been reported that targeting ligands on the surface of liposome leads to the binding, close apposition, and fusion of the lipid layers with the targeted cell lipid membrane [18,19]. Subsequently, the contents of liposome enter into the cells. We also noticed that there were more uptake of 800CW in the endothelial cells than the U87 tumor cells post a single dose of 6 Gy (Figure 2). The data are consistent with the aforementioned study by He *et al.* [9], indicating that U87 tumor cells are radioresistant to radiation compared to vascular endothelial cells.

### 2.3.*In vivo* Longitudinal NIR Optical Imaging of PGN-L-800CW in U87 Glioma Xenografts

**Figure 3:**
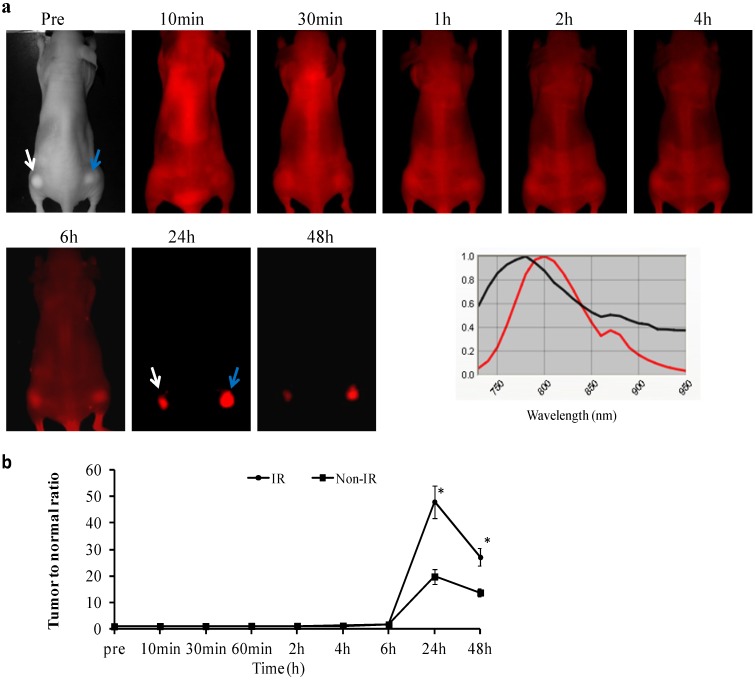
*In vivo* longitudinal NIR imaging of exposed PS in gliomas with/without radiation. (**a**) A mouse bearing representative size-matched subcutaneous U87 tumors (arrows) on each thigh received 12 Gy of irradiation to the right side tumor (blue arrow). Twenty-four hours after radiation, PGN-L-800CW (1.8 nmol/mouse) was injected via a tail vein and a series of *in vivo* fluorescence images was acquired at different time points. There were significant normal tissues signals during the first 6 h post injection, indicating that the nanoprobes were remaining in blood circulation. However, the optical signals were only detected from the tumors at 24 h, which sustained at least to 48 h post injection. Representative *in vivo* emission wavelengths of the PGN-L-800CW (red) and normal tissue fluorescence (black) were presented (**b**) Time course curves of TNR revealed a maximal TNR = 20 ± 3 at 24 h for the non-irradiated tumors (*n* = 6). Irradiation of the tumors (*n* = 6) increased levels of exposed PS to a TNR of 48 ± 6 (* *p* < 0.05 from non-IR).

**Figure 4:**
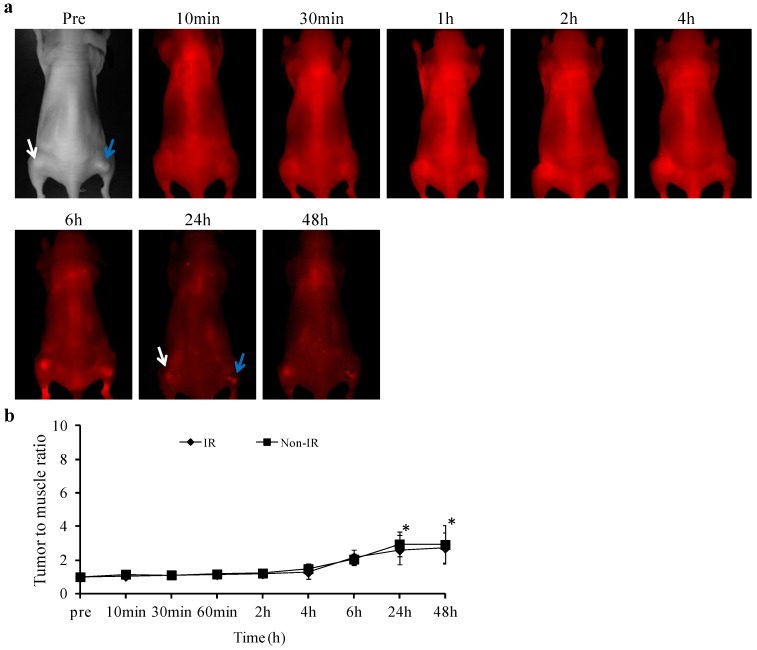
*In vivo* NIR imaging of Aur-L-800CW. (**a**) The control liposomal nanoprobes, Aur-L-800CW were injected into the mice (*n* = 3) 24 h after irradiating one of the tumors on the thighs (blue arrow). A series of whole body images was acquired. Certain tumor accumulations of the probes were seen at later time points; (**b**) Quantitative analysis of TNR indicated significantly increased TNR in both the non-irradiated (white arrow) and irradiated tumors (* *p* < 0.05), implicating the EPR effect causing tumor retentions of the control probes.

*In vivo* NIR imaging detected no significant uptake of PGN-L-800CW during the first 6 h post *i.v.* injection in either the non-irradiated tumors (*n* = 6) or the irradiated tumors (*n* = 6), as presented in Figure 3. However, NIR imaging revealed clear tumor contrast, with essentially no normal tissue background at 24 h post injection. Mean tumor/normal ratio (TNR) of light intensity was 20 ± 3 for the non-irradiated tumors. We have previously demonstrated that 27% tumor vascular endothelial cells of U87 gliomas have exposed PS, which is increased to 64% after a single dose of 12 Gy radiation. Indeed, significantly enhanced tumor contrast was observed for the irradiated tumors at 24 h post injection of PGN-L-800CW (mean TNR = 48 ± 6, *p* < 0.05; Figure 3b). TNR in both of the tumors declined afterwards, but still remained significantly high at 48 h post injection (mean = 12 ± 1 and 25 ± 3, respectively; Figure 3). As compared to the PGN-800CW probes used in our previous study, the liposomal nanoprobe, PGN-L-800CW achieved >10 fold increase in tumor contrast [10]. The large payload of 800CW dye within the liposome and increased binding affinity owing to a large number of surface antibodies likely contribute to the enhanced signals. Moreover, appearance of tumor contrast with PGN-L-800CW was somewhat later than that with PGN-800CW. The clear tumor contrast was visualized in the irradiated tumors as early as 4 h post injection of PGN-800CW in our previous study, while it was not seen until 24 h post PGN-L-800CW in the current study. The discrepancy may result from the longer circulation time of the liposomal nanoprobes. We further studied the *in vivo* binding specificity of PGN-L-800CW by administering the control antibody labeled liposomal nanoprobe, Aur-L-800CW into the U87 glioma bearing mice. In contrast to the minimal tumor accumulation observed for the control Aur-800CW probes in our previous study [10], optical imaging revealed significantly increased tumor contrast in both the non-irradiated and irradiated tumors at 24 h post injection of Aur-L-800CW (mean TNR = 2.6 ± 1 and 2.9 ± 1, respectively; *p* < 0.05; Figure 4). The data, at first sight, seem contradictory, not supportive of the tumor targeting specificity of PGN-L-800CW. The results are actually in good agreement with those reported by others [19]. Intratumoral accumulations of the non-targeted liposomes are believed to associate with the enhanced permeability of tumor vasculature and the prolonged circulation time of liposomes, also known as the EPR effect. Nevertheless, the EPR effect could account for only a small fraction of the tumor-specificity of PGN-L-800CW since its TNR was 16-fold higher than that of the control Aur-L-800CW (Figure 3 and Figure 4). Moreover, recent studies by others have also noticed that the EPR effect varies significantly both intra- and inter-tumorally due to heterogeneous nature of tumors [20,21]. Therefore, functionalization of liposomes with tumor-specific targeting moieties will be essential to achieve enhanced tumor-targeting specificity and sensitivity [21,22,23]. In particular for the real situation of glioma growing orthotopically, delivery of imaging contrast or drugs to parenchymal tumor tissue encounters the biological barrier, the blood brain barrier (BBB). Despite the fact that the BBB becomes disrupted with tumor growth, there are still significant portions of intratumoral regions that maintain the intact BBB. Thus, it could be advantageous for PS-targeted delivery that targets the luminal surface, lacks a need to penetrate the BBB. However, future studies using orthotopic glioma models will be critical for assessing the targeting specificity and sensitivity of PGN-L-800CW.

Cell surface biomarkers, in particular, those on tumor vascular endothelial cells, are attractive targets because they are the most readily accessible target sites. Antibodies, antibody derivatives or peptides against tumor vascular specific antigens have been extensively studied for their ability to facilitate cancer molecular imaging. Applications of αvβ3 binding ligands, such as arginine-glycine-aspartic acid (RGD)-containing peptides have been successful in tumor imaging. RGD-based imaging probes have been developed for optical imaging, PET, SPECT and MRI of tumor angiogenesis in both preclinical and clinical settings [24,25,26]. In this study, we have utilized a fully human monoclonal antibody, PGN635, to target PS exposed on tumor vascular ECs. PS is strictly located in the inner leaflet of the plasma membrane bilayer in normal cells, including the vascular endothelium. Cell surface-exposed PS is therefore an attractive target for tumor molecular imaging. PGN635 recognizes PS in a β2GP1-dependent fashion. PGN635 and related antibodies have a more restricted specificity for PS than does annexin V, which recognizes PE in addition to PS and other anionic phospholipids. [4,27,28] This is of clinical importance because non-invasive molecular imaging of apoptosis could give an early indication of the responsiveness of a patient’s tumor to therapy, allowing alterations in the therapy to be made if the responses were not as good as expected. Given the high binding affinity and specificity to PS, PS-targeting antibodies*,* such as PGN635 could be a better alternative to develop prognostic imaging probes to monitor treatment response.

### 2.4. *Ex vivo* Imaging of Biodistribution of PGN-L-800CW

**Figure 5:**
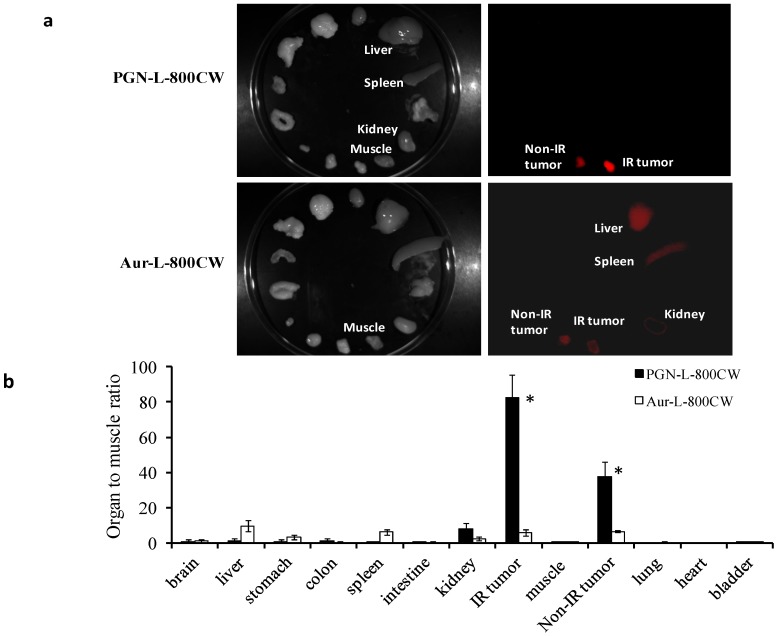
*Ex vivo* optical imaging of biodistribution of the liposomal nanoprobes. (**a**) Immediately after *in vivo* optical imaging at 48 h, *ex vivo* optical imaging was performed and average photon counts were obtained for tumors and normal organs and normalized by the muscle value; (**b**) For the mice injected with PGN-L-800CW, both the non-irradiated (*n* = 5) and irradiated (*n* = 5) tumors had significantly higher TMR (mean = 38 ± 8 and 83 ± 13, respectively), while the highest TMR among the normal organ/tissue was obtained from kidney (mean = 8 ± 3). Liver and spleen had a minimal accumulation. By contrast, significant amounts of the control Aur-L-800CW probes (*n* = 3) were found in liver (TMR = 9) and spleen (TMR = 6). Both the non-irradiated and irradiated tumors had a mean TMR = 6.

*Ex vivo* NIR imaging was conducted on the excised tumors and normal tissues and organs immediately after *in vivo* imaging at 48 h post administration of the liposomal probes. For the PGN-L-800CW group (*n* = 5), optical signal was seen exclusively from the non-irradiated and irradiated tumors with minimal background signal from normal tissues, as shown in Figure 5.

Mean tumor/muscle ratio (TMR) for the irradiated and non-irradiated tumors was 83 ± 13 and 38 ± 8, respectively (Figure 5). Lack of significant accumulation of PGN-L-800CW in liver or spleen was quite unexpected because liposomes are known to be cleared primarily by the reticuloendothelial system (RES) of liver and spleen. This further suggests that the highly tumor-specific binding of PGN-L-800CW may have altered their pharmacokinetics and biodistribution. Interestingly, kidney had the notably higher TMR (mean = 9) than that of liver or spleen. The hydrophilic 800CW dye is expected to undergo renal excretion after release from liposomes due to endocytosis. By contrast, large amounts of the control Aur-L-800CW (*n* = 3) were detected in liver and spleen (TMR = 9 and 6, respectively), as compared to a TMR of 6 for both the non-irradiated and irradiated tumors. Again, the data implicate that the EPR effect plays an important role on intratumoral accumulation of the non-specific probes.

### 2.5. Histological and Immunohistochemical Analysis

**Figure 6:**
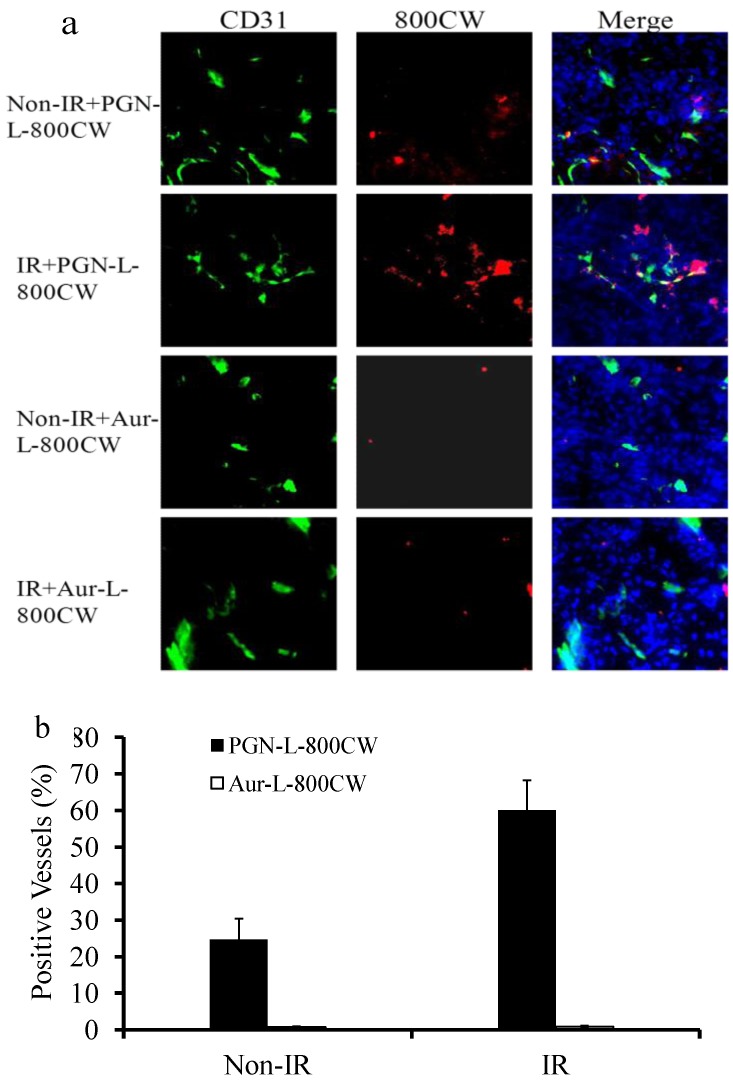
Fluorescence microscopic detection of the liposomal nanoprobes in non-irradiated or irradiated gliomas. Fluorescence microscopy with NIR filters was applied to detect 800CW signals. (**a**) Frozen sections of non-irradiated or irradiated tumors were examined for the presence of CD31 (green), 800CW (red), or DAPI (blue). Significantly more 800CW signals were observed in the PGN-L-800CW treated tumors than that in the control Aur-L-800CW tumors. Irradiation induced more PGN-L-800CW bound to exposed PS. Co-localizations of 800CW with tumor vascular endothelial cells (CD31) were primarily observed for PGN-L-800CW; (**b**) Quantitative data showed that 25% ± 6% of non-irradiated tumor vascular endothelial cells were co-stained with PGN-L-800CW, which increased to 60% ± 8% after irradiation. By contrast, minimal Aur-L-800CW signals were detected from either non-irradiated (0.7% ± 0.1%) or irradiated (0.9% ± 0.2%) tumors.

Fluorescence microscopy confirmed abundant 800CW signals in the non-irradiated and irradiated tumors of the mice injected with PGN-L-800CW (Figure 6).

Both intravascular signals, co-localizing with tumor vascular endothelial cells (anti-CD31) and extravascular signals were seen. Twenty five percent of tumor vascular endothelial cells (25% ± 6%) of the U87 tumors without irradiation had intrinsic PS exposure that was bound to PGN-L-800CW, while significantly enhanced PS exposure (60% ± 8%) on tumor blood vessels was evidenced after a single dose of 12 Gy irradiation (*p* < 0.05; Figure 6b). Significantly fewer NIR signals were seen in the tumors injected with the control Aur-L-800CW, further supporting the specificity of tumor-targeted PGN-L-800CW (*p* < 0.01; Figure 6b). These data were in good agreement with our previous quantitative data of PS exposure on tumor vasculature of U87 gliomas, determined by using the full length PGN635 [10].

## 3. Experimental

### 3.1. Preparation and Characterization of PS-Targeted Liposomes Loaded with Optical Imaging Contrast

The procedure used for preparation of liposomes has been described previously [29]. In brief, egg phosphatidylcholine (EPC) (Sigma-Aldrich Corporation, St. Louis, MO, USA), cholesterol (Sigma-Aldrich Corporation), 1,2-distearoyl-sn-glycero-3-phosphoethanolamine-*N*-[methoxy(polyethylene glycol)_2000_] (PEG_2000_–DSPE), and 1,2-distearoyl-sn-glycero-3-phosphoethanolamine-*N*-[carboxy-(polyethylene glycol)_2000_] (COOH-PEG_2000_-DSPE, Avanti Polar Lipids, Alabaster, AL, USA) were dissolved in chloroform (57:51:4:1 μmol/μmol) in a pear-shaped flask. The lipid film was prepared by removing the chloroform using a rotary vacuum evaporator. The rough 800CW liposomes were produced by hydration of the film with PBS containing IRDye 800CW (LI-COR Biosciences, Lincoln, NE, USA) with sonication in water bath for 5 min, followed by sonication using a probe-type sonicator (Omni International Inc, Kennesaw, GA, USA) for 5 min. The 800CW liposomes were then extruded through polycarbonate membranes (400 nm and 200 nm pore sizes, respectively, Nalgene, Rochester, NY, USA). The sample was dialyzed (6,000–8,000 molecular mass cutoff) in the PBS to remove free 800CW. The content of 800CW on the liposomes was determined by a microplate reader (Molecular Devices, Sunnyvale, CA, USA).

To functionalize the bimodal liposomes with PS-targeting antibody, the human monoclonal antibody PGN635 that was generated by Affitech A.S. (Oslo, Norway) in collaboration with Peregrine Pharmaceuticals. Inc., (Tustin, CA, USA) was used. Aurexis is a human monoclonal antibody that binds to an irrelevant antigen (*S. aureus* clumping factor A) and was used as a negative control antibody. PGN635 and Aurexis F(ab’)_2_ fragments were generated by reacting antibodies with pepsin at a molar ratio of 1:130 (antibody:pepsin) for 1 h at 37 °C. F(ab’)_2_ fragments (MW = 110 kD) were purified by FPLC using an S*-200* column (Pharmacia, Piscataway, NJ, USA) and PBS running buffer. PGN635 F(ab’)_2_ or aurexis F(ab’)_2_ were then conjugated to the distant terminus of polyethylene glycol (PEG) chain-coated liposomes. Briefly, 3.55 μmol of 1-(3-dimethylaminopropyl)-3-ethylcarbodiimide hydrochloride (EDC, Sigma-Aldrich) and 7.92 μmol of *N*-hydroxysuccinimide (NHS, Sigma-Aldrich) were added into 2 mL of 800CW liposomes (COOH-PEG_2000_-DSPE:EDCI:NHS = 0.02:3.55:7.92, μmol/μmol) and gently stirred for 30 min at room temperature. PGN635 F(ab’)_2_ or aurexis F(ab’)_2_ (0.01 μmol) were then added into the suspensions and allowed to continuously react for 5 h at room temperature. Excess EDC and NHS were removed by dialysis. The liposome suspensions were applied to a quick spin Sephadex G-50 column (Fisher Scientific, Pittsburg, PA, USA) equilibrated with PBS and centrifuged at 150 *g* at 4 °C to remove the uncoupled antibodies. Subsequently, liposome fractions were collected from quick spin Sephadex G-50 column, and the content of antibodies on the liposomes was determined by the bicinchoninic acid (BCA) kit (Sigma-Aldrich). The nanoprobes are referred to as PGN-L-800CW or the control, Aur-L-800CW.

The mean diameter and zeta potential of the liposome nanoprobes were measured by dynamic light scattering (DLS) analysis with Zetasizer 3000HSA (Malvern Instruments Ltd., Malvern, UK). The data were presented in Table 1.

### 3.2. Stability of PGN-L-800CW

*In vitro* release of 800CW in the liposomes was performed by the dialysis against the release medium containing serum protein (phosphate buffered saline containing 10% fetal calf serum). A volume of 2.0 mL liposomes plus 2.0 mL of release medium in dialysis tubing was immersed in 20.0 mL of the release medium, and oscillated with a shaker at a rate of 100 times per minute at 37 °C. A volume of 0.5 mL release medium was taken at 0, 0.25, 0.5, 1, 2, 4, 6, 8, 12, 24 and 48 h, respectively, and immediately replaced with the same volume of fresh release medium after each sampling. The 800CW content in the release medium was determined by the microplate reader. The release rate was calculated with the formula: RR = (Wi/W_total_) × 100%, where RR is the drug release rate (%), W_i_ is the measured amount of 800CW at the time-point of ith h in release medium, and W_total_ is the total amount of 800CW in the equal volume of liposome suspensions prior to dialysis.

### 3.3. *In Vitro* Cytotoxicity

Adult bovine aortic endothelial cells (ABAE, Clonetics, Walkersville, MD, USA) were cultured in DMEM medium (Sigma-Aldrich) supplemented with 10% fetal bovine serum (Invitrogen, Grand Island, NY, USA), 100 units/mL penicillin (Sigma-Aldrich), and 100 units/mL streptomycin (Sigma-Aldrich). Cytotoxicity of PGN-L-800CW was assessed by using the trypan blue staining assay. ABAE cells were seeded into 6-well culture plates at a density of 2.8 × 10^5^ cells per well and grown in culture medium in the incubator at 37 °C. PGN-L-800CW or the control Aur-L-800CW was added into 6-well culture plates containing 800CW of 0–200 µg/mL, respectively. The survival rate was determined at 48 h by 0.4% trypan blue staining, and the number of viable cells were measured by an automated cell counter (Bio-Rad, Hercules, CA, USA).

### 3.4. *In Vitro* Binding Specificity

ABAE cells and human glioma U87MG cells (ATCC, Manassas, VA, USA) were used. To induce PS exposure, the cells were treated with a single dose of 6 Gy X-radiation. Twenty-four hours later, the irradiated cells were incubated with PGN-L-800CW or the control Aur-L-800CW at a concentration of 2.0 µg/mL 800CW for 1 h. For the blocking study, the cells were pretreated with the full length PGN635 for 1 h prior to PGN-L-800CW. Unbound liposomes were washed away with PBS, and then the cells were fixed with 4% paraformaldehyde (PFA). Cell membrane and cytoskeleton were stained with green fluorescence labeled phalloidin (Invitrogen) and nuclei were counterstained by 4',6-diamidino-2-phenylindole (DAPI). The NIR fluorescence signal of PGN-L-800CW or Aur-L-800CW was detected using a Zeiss AxioObserver (Carl Zeiss MicroImaging, Inc., Thornwood, NY, USA) equipped with NIR filters. The NIR signals were recorded and merged with the phalloidin and DAPI images.

### 3.5. Glioma Xenografts in Nude Mice

All animal procedures were approved by the Institutional Animal Care and Use Committee of University of Texas Southwestern Medical Center. Human glioma U87MG cells in 100 μL of serum-free medium containing 25% Matrigel (BD Biosciences, San Jose, CA, USA) were injected subcutaneously on the both thighs of anesthetized nude mice (*n* = 9; BALB/c nu/nu, 6–8 weeks old; NCI, Frederick, MD, USA).

### 3.6. *In Vivo* Near Infrared Optical Imaging of Tumor Targeted PGN-L-800CW

*In vivo* NIR optical imaging was performed using a Maestro imaging system (CRi, Inc, Woburn, MA, USA). When the subcutaneous tumors on both thighs reached ∽5 mm in diameter, a single dose of 12 Gy of irradiation was delivered to the tumors on the right thigh of anesthetized mice using a small animal irradiator (XRAD320, Precision X-ray, Inc. North Branford, CT, USA) fitted with a variable collimator to generate a single adjustable collimated iso-dose beam of X-rays at a dose rate of 10 Gy/min. Twenty-four hours later, each mouse was anesthetized and maintained under general anesthesia (air and 2% isoflurane). NIR images were acquired before and at different times after administration of PGN-L-800CW (*n* = 6, 1.8 nmol/mouse) or the control Aur-L-800CW (*n* = 3, 1.8 nmol/mouse) through a tail vein. For optical imaging analysis, average photon counts normalized by time (s) in tumors and adjacent normal skin were obtained. Tumor/normal ratio (TNR) was used for quantifying dynamic changes in signal intensity, as previously described [30].

### 3.7. *En Vivo* Optical Imaging of Biodistribution

Immediately after *in vivo* imaging at 48 h, the tumor-bearing mice were sacrificed. Major organs and tumor tissues were dissected and subjected to optical imaging (Maestro, CRi, Inc, Woburn, MA, USA). Average photon counts (photons/s/m^3^) was obtained for tumors and each organ/tissue and normalized by the muscle value.

### 3.8. Immunohistochemical Studies

The cryosections of tumors were immunostained with antibodies to the endothelial marker, CD31 (Serotec, Raleigh, NC, USA) followed by Cy2-conjugated secondary antibody (Jackson Immunoresearch Laboratories, West Grove, PA, USA). The NIR fluorescence signal was detected using the Zeiss AxioObserver equipped with NIR filters. The NIR signals were recorded and merged with the CD31 image and the DAPI-stained image of the same field.

### 3.9. Statistical Analysis

Statistical significance was assessed using an ANOVA on the basis of Fisher’s protected least significant difference (PLSD; Statview; SAS Institute Inc., Cary, NC, USA) or Student’s t tests.

## 4. Conclusions

In summary, we have developed a PS-targeted liposomal nanoprobe, PGN-L-800CW, for near infrared optical imaging and investigated its binding specificity and *in vivo* sensitive tumor targeting. In comparison with our previous study of direct conjugates of PGN635 and IRDye^®^ 800CW, the liposomal nanoprobe showed superior tumor targeting characteristics, enabling enhanced *in vivo* imaging of U87 gliomas in mice. Clear visualization of cytoplasm localization of PGN-L-800CW indicates internalization of the probes, which is distinct from previous observations of the external cell membrane localization of the antibody complex or the 800CW-PGN. Irradiation significantly increased PS exposure in tumor vascular ECs and tumor cells, resulting in enhanced tumor contrast by optical imaging. Significantly reduced liver and spleen uptake further suggest the promising pharmacological property of the PGN-L-800CW nanoprobes. Utilizing this PS-targeted liposomal platform, both imaging contrast agents and chemotherapeutics can be encapsulated to achieve tumor diagnosis, imaging-guided drug delivery and potentially non-invasive monitoring of treatment simultaneously.
